# Post-COVID-19 Arthritis and Sacroiliitis: Natural History with Longitudinal Magnetic Resonance Imaging Study in Two Cases and Review of the Literature

**DOI:** 10.3390/v13081558

**Published:** 2021-08-06

**Authors:** Donatella Colatutto, Arianna Sonaglia, Alen Zabotti, Lorenzo Cereser, Rossano Girometti, Luca Quartuccio

**Affiliations:** 1 Clinic of Rheumatology, Azienda Sanitaria Universitaria del Friuli Centrale, Piazzale Santa Maria della Misericordia 15, 33100 Udine, Italy; donatella.colatutto@gmail.com (D.C.); arianna.sonaglia23@gmail.com (A.S.); alen.zabotti@asufc.sanita.fvg.it (A.Z.); 2 Department of Medicine (DAME), University of Udine, Via Colugna 50, 33100 Udine, Italy; rossano.girometti@uniud.it; 3 Institute of Radiology, Azienda Sanitaria Universitaria del Friuli Centrale, Piazzale Santa Maria della Misericordia 15, 33100 Udine, Italy; lorenzo.cereser@asufc.sanita.fvg.it; 4 Rheumatology Clinic, Department of Medicine (DAME), University of Udine, ASUFC, Via Colugna 50, 33100 Udine, Italy

**Keywords:** sacroiliitis, arthritis, COVID-19, post-COVID-19 manifestations

## Abstract

Severe acute respiratory coronavirus-2 syndrome (SARS-CoV-2) is a well-known pandemic infectious disease caused by an RNA virus belonging to the coronaviridae family. The most important involvement during the acute phase of infection concerns the respiratory tract and may be fatal. However, COVID-19 may become a systemic disease with a wide spectrum of manifestations. Herein, we report the natural history of sacroiliac inflammatory involvement in two females who developed COVID-19 infection with mild flu-like symptoms. After the infection they reported inflammatory back pain, with magnetic resonance imaging (MRI) studies showing typical aspects of sacroiliitis. Symptoms improved with NSAIDs therapy over the following months while MRI remained positive. A literature review was performed on this emerging topic. To our knowledge, this is the first MRI longitudinal study of post-COVID-19 sacroiliitis with almost one year of follow-up. Predisposing factors for the development of articular involvement are unclear but a long-lasting persistence of the virus, demonstrated by nasopharyngeal swab, may enhance the probability of altering the immune system in a favourable background.

## 1. Introduction

In December 2019, a new type of pneumonia emerged, caused by COVID-19, a member of coronaviridae family. The initial outbreak took place in Wuhan, but rapidly diffused worldwide, such that the World Health Organisation (WHO) declared the disease a pandemic in March 2020 [1,2,3,4].

Compared to the previous severe acute respiratory syndrome-related coronavirus (SARS-CoV) and the Middle East respiratory syndrome-related coronavirus (MERS-CoV), SARS-CoV-2 is much more transmissive and dangerous, with a wide spectrum of systemic manifestations besides respiratory symptoms [1,2,5,6].

The disease caused by SARS-CoV-2 is often characterised by a dry cough, fever, dyspnoea, fatigue, general malaise, anosmia, ageusia, a sore throat, diarrhoea, nausea and vomiting. However, other symptoms have been reported in the literature [1,5,6,7,8,9,10,11,12].

Regarding articular involvement, some cases of arthritis have been described after SARS-CoV-2 infection, as could happen in other viral diseases, but further studies and long-term follow-up are required [13,14,15,16,17,18,19,20,21,22,23,24,25,26,27,28,29,30,31,32,33,34,35,36,37,38,39].

Articular involvement in COVID-19 can occur in different stages of the disease and it may be represented by non-specific arthralgia or by acute arthritis. These manifestations generally respond to non-steroid anti-inflammatory drugs (NSAIDs), but sometimes steroid treatment is required [15,16,23].

Sometimes patients can develop other musculoskeletal symptoms and signs, such as enthesitis, tenosynovitis or dactylitis [13,14,15,16,17,18,20,21,22,24,27,29,30,31,32,33,34,35,36,37,38,39]; isolated reactive inflammatory sacroiliac involvement after COVID-19 has been rarely recorded.

## 2. Case Reports

Herein, we report the natural history and long-term follow-up of two cases of sacroiliitis that developed after COVID-19 infection. They were two females aged 58 and 53, both working as healthcare professionals in a nursing home with old patients. They developed SARS-CoV-2 infection in March 2020 after work exposure, and were referred to our Rheumatologic Clinic for musculoskeletal symptoms such as inflammatory joint pain and myalgia, which started shortly after SARS-CoV-2 infection.

Demographic/clinical characteristics are illustrated in Table 1, while Table 2 describes laboratory tests at SARS-CoV-2 infection diagnosis and during the patients’ follow-up.

**Figure 1:**
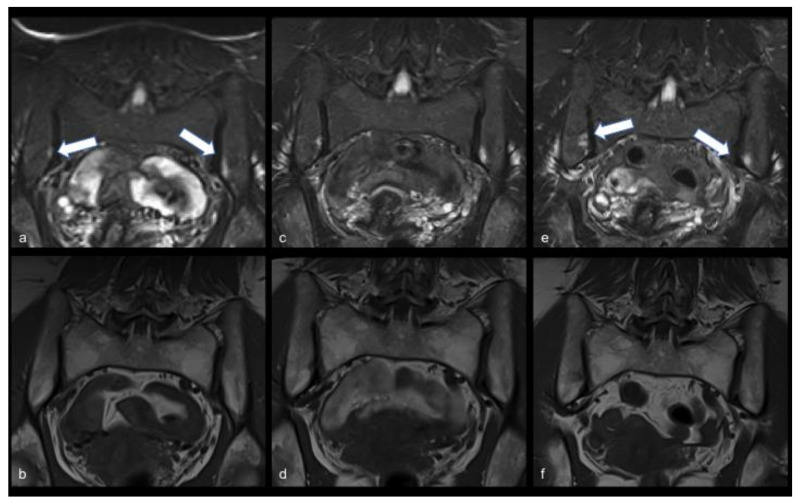
Case report 1. Persistent MRI findings suggestive of bilateral active sacroiliitis in a 58-year-old patient with lower back pain. On the first MRI examination, subchondral bone marrow oedema at the iliac side of both sacroiliac joints was found, appearing as band-like hyperintensity on short-tau-inversion recovery (STIR) T2-weighted semi-coronal image (arrows in (**a**)) with corresponding hypointensity on turbo-spin-echo (TSE) T1-weighted semi-coronal image (**b**). Oedema was found to persist at a second MRI examination performed one month later (**c**,**d**), and increase after six more months (**e**,**f**), especially on STIR T2-weighted image (arrows in (**e**)).

**Figure 2:**
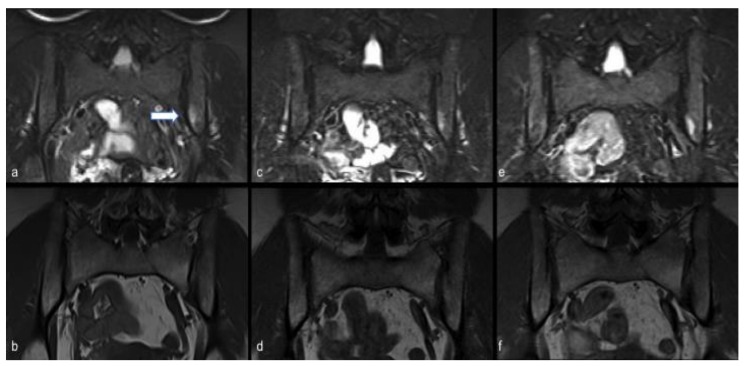
Case report 2. Persistent MRI findings of unilateral active sacroiliitis in a 53-year-old woman with lower back pain. At the time of first MRI examination, subchondral bone marrow oedema at the iliac side of the left sacroiliac joint was observed, appearing as band-like hyperintensity on short-tau-inversion recovery STIR T2-weighted semi-coronal image (arrow in (**a**)) with corresponding hypointensity on turbo-spin-echo TSE T1-weighted semi-coronal image (**b**). Oedema was found to persist at subsequent MRI examinations performed after one month (**c**,**d**) and six months (**e**,**f**).

Finally, complete magnetic resonance imaging (MRI) follow-up is shown in Figure 1 and Figure 2 for case report 1 and 2, respectively.

### 2.1. Case Report 1

At the end of March 2020, a 58-year-old woman complained of a dry cough without dyspnoea or fever and tested positive for a SARS-CoV-2-nasopharyngeal swab on March 27. She repeated the nasopharyngeal swab test several times until the first negative result, which occurred on April 24. Because of persistent positive swabs, she was treated with HCQ and azithromycin; on May 22 the patient performed a SARS-CoV-2 serological test showing negative IgM and slightly positive IgG, with limited sensibility and specificity. The patient suffered from autoimmune hypothyroidism treated with levothyroxine without other chronic therapy. She had no familiar history for rheumatologic diseases, psoriasis or IBD. 

At the end of April, she complained of inflammatory arthralgia and myalgia, especially in both shoulder and pelvic girdles, and bilateral buttock pain (right > left) with persistent dry cough. The patient reported that inflammatory pain was slowly and spontaneously improving from the end of May 2020 in the absence of chronic NSAID administration or other drugs.

At the end of May, upon physical examination she had bilateral sacroiliac joint pain without peripheral joint tenderness or swelling and no evocable tender points. Chest and abdominal examinations were normal.

Blood samples showed no inflammation and a mild elevation of CPK.

Extensive laboratory tests were performed, including cytokine profile, which did not provide further information regarding systemic inflammation (Table 1). Autoantibodies were negative. The patient presented HLA-B8 and -B57, while HLA-B27 was negative. The first MRI of shoulders and pelvic girdle (20 June 2020) showed bilateral sacroiliitis with bone marrow oedema, particularly on the left side (Figure 1a,b). Neither signs of bone damage nor alterations of the muscles were found.

In July 2020, the repeated MRI showed the same alteration as the first one (Figure 1c,d). She reported further improvement in articular and muscle pain but persistence of the same inflammatory back pain and at the clinical examination of sacroiliac joints, tenderness was still present.

Serological test for SARS-CoV2 was repeated, showing positive IgG and borderline IgM.

Blood samples were also repeated and no inflammation was found, but a mild elevation of CPK was confirmed. Serum cytokine profile was substantially normal, showing only a very mild increase in IL2R and TNF alpha at the second follow-up visit.

Because of sacroiliac joint pain, NSAIDs therapy was started in July for 10 days.

She was evaluated again in November 2020, reporting clinical recovery with only use of NSAIDs as needed.

MRI was repeated in January 2021 and it showed increased bone marrow oedema of the sacroiliac joints (Figure 1e,f). On physical examination, the patient still complained about mild low back pain. She continued to sporadically use NSAIDs, as needed. Blood tests showed no inflammation and normalisation of CPK.

### 2.2. Case Report 2

A 53-year-old female, working as a healthcare professional in the same nursing home as the previous patient, developed a dry cough without fever at the end of March 2020 and tested positive after repeated SARS-CoV-2 nasopharyngeal swabs (the first on March 27). She was treated with HCQ and azithromycin with the first negative result of the swab on April 24. A SARS-CoV-2 serological test performed on May 2020 showed negative IgM and slightly positive IgG.

In the middle of April, she developed very similar symptoms to her colleague, with a polymyalgic symptoms associated with inflammatory back pain.

In May, blood tests showed mild systemic inflammation (CRP 19 mg/L, normal values below 5 mg/L) and CPK elevation (412 IU/L), but they normalised one month later. Serum cytokine profile revealed only a very mild increase in IL8 at the first follow-up visit.

She stated that these symptoms were gradually and spontaneously improving from the end of May without any treatment. On clinical examination she had mild tenderness of the sacroiliac joints. At the MRI performed on June 3rd, bone marrow oedema suggestive of sacroiliitis was found (Figure 2a,b). Serologic test for SARS-CoV-2 showed positive IgG and negative IgM. After blood tests, no inflammation was found, with normalisation of CRP and CPK. Considering the patient’ symptoms and MRI findings, NSAIDs therapy was initiated with subjective improvement. She repeated the MRI on July 3rd and it was unchanged (Figure 2c,d).

At the examination in November 2020, she was using NSAIDs occasionally.

At the follow-up in January 2021 she reported mechanical low back pain and occasional use of NSAIDs, with mild tenderness of the sacroiliac joints upon physical examination; the MRI performed on February 2021 showed unchanged bone oedema and blood tests were normal (Figure 2e,f).

## 3. Materials and Methods

We conducted a PubMed search utilising the keywords “post-COVID-19 arthritis”, “post-COVID-19 sacroiliitis”, “reactive arthritis”, “reactive sacroiliitis”, “acute sacroiliitis”, “post-COVID-19 manifestations” and “post-COVID-19 rheumatic diseases”. About one hundred articles were evaluated and the most relevant were reviewed with more attention; significant data pertinent to the aim of our study were extracted and organised. In particular, the most significant articles were represented by reviews concerning reactive arthritis, post-viral arthritis, acute sacroiliitis and post-COVID-19 rheumatologic manifestations; case reports about post-COVID-19 arthritis were also considered.

Serum IL-6 (pg/mL) was measured by CE-IVD electrochemiluminescence immunoassay (Elecsys IL6, Cobas, Roche, Basel, Switzerland; physiological range < 7 pg/mL). All other soluble factors were quantified in the same sera of patients with a magnetic bead-based multiplex assay (Bio-Plex Pro™ Custom Human Cytokines and Chemokine Panel, procarta-Plex, Bio-Rad Laboratories, Hercules, California) according to the manufacturer’s instructions. All dosages were obtained in sera stored in aliquots at −80 °C.

## 4. Results

Case reports of supposed SARS-CoV-2-related arthritis are summarised in Table 3 [13,14,15,16,17,18,20,21,22,24,27,29,30,31,32,33,36,37,38].

We also included in our review case reports from Novelli and Baimukhamedov [34,35]. The first described the case of a 27-year-old woman who developed psoriatic spondyloarthritis triggered by SARS-CoV-2 infection. The patient developed peripheral arthritis and mild bilateral sacroiliitis confirmed on MRI; she was HLA-B27 negative and had a family medical history of psoriasis [34]. Baimukhamedov et al. reported the development of chronic arthritis, which fulfilled the classification criteria for seropositive rheumatoid arthritis, one month after COVID-19 infection [35].

Table 3 describes the characteristics of 19 cases, 12 males and 7 females, who were diagnosed as affected by SARS-CoV-2-related arthritis, without fulfilling the classification criteria for other arthritis.

The onset of the articular symptoms occurred two to four weeks after the infection in the majority of cases, while in two cases articular symptoms rose concomitantly to the diagnosis of COVID-19. The pattern of arthritis was usually mono-oligoarticular, mainly in the lower limbs (11/19), was polyarticular in 6/19 cases, one case had only extensor tendonitis of the right hand and another patient developed COVID-19-related dactylitis. Synovial fluid was negative for crystals. Autoantibodies, rheumatoid factor and antinuclear antibody were negative in all patients in whom these analyses were performed. HLA B27 was positive in one case.

Imaging study was not available in all patients. Five patients were evaluated by X-rays which revealed no erosions in all, while five patients were evaluated by ultrasound (US), which revealed synovitis, and three patients were studied by MRI, which showed signs of inflammation.

In some of these cases, synovial fluid analysis was not performed, so we cannot exclude the presence of crystals. Lopez Gonzalez et al. reported four cases of crystal-induced arthritis (MSU and CPP crystals detected with polarised light microscopy of the synovial fluid) during hospitalisation for SARS-CoV-2 infection [23,28].

Alivernini et al. also performed synovial biopsy in two patients, but in one of them an exacerbation of RA arthritis during SARS-CoV-2 infection was diagnosed, so we did not include this case [30].

Most of those cases resolved with NSAIDs or with corticosteroids (administered orally or with intra-articular injection). One case was treated with topical NSAIDs, oral opioid and gabapentin, one case was treated with baricitinib and corticosteroids and, finally, one case did not require any treatment.

Sacroiliac joint involvement after COVID-19 infection was reported in three cases, [34,38,39]; all of them fulfilled the classification criteria for psoriatic arthritis [34], or HLA-B27-positive axial spondyloarthritis [38,39]. Moreover, in the case report by Coath et al., COVID-19 infection was not confirmed by nasopharyngeal swab [39].

Notably, the case reported by De Stefano et al. presented transient psoriatic skin lesions, and in the patient treated by Cincinelli et al., nail psoriasis was previously diagnosed [31,37]. However, the authors did not conclude a psoriatic arthritis diagnosis with sufficient certainty in either case.

Finally, Salvatierra et al. described a case of SARS-CoV-2-related dactylitis in a sixteen-year-old girl that resolved without NSAIDs [33].

## 5. Discussion

Articular involvement in SARS-CoV-2 infection may occur at different times, since it could be an initial symptom of the infection, emerge during the acute phase (sometimes during hospitalisation) or manifest after recovery.

When it appears during the active phase of the infection, it is generally represented by non-specific arthralgia, while it could develop into acute arthritis when arising in the healing period; this feature deviates from classic viral-related arthritis, where joint involvement usually occurs during the viremia period with acute manifestations of the infection, as observed by Gasparotto and Ono, and assimilates this clinical manifestation to post-infectious arthritis [14,18].

The relationship with HLA-B27 positivity did not emerge for COVID-19-related arthritis, while its influence on reactive arthritis is well known.

Since 1999, the term “reactive arthritis” can be applied only to the clinical picture and the microbes associated with HLA-B27 and spondyloarthritis [40]. Thus, the term post-COVID-19 sacroiliitis is more appropriate.

Post-COVID-19 arthritis may present with mono- or oligoarticular involvement, with a predilection for lower limb joints. Generally, it begins some days or a few weeks after the resolution of other manifestations of the infection [13,14,15,16,17,18,20,21,22,24,27,29,30,31,32,33,36,37,38,39]. As observed by Di Carlo et al., other possible features of post-viral arthritis are represented by enthesitis, dactylitis and tenosynovitis [17,33].

Post-COVID-19 arthritis generally responds to NSAIDs, prolonged for 2–4 weeks, like other viral arthritis. However, steroid treatment is sometimes required, preferably administered with intra-articular injection (if there is mono-oligoarticular involvement); in a few cases, systemic steroid is necessary, but generally for short periods [13,14,15,16,17,18,20,21,22,24,27,29,30,31,32,33,36,37,38,39].

Only a few cases of SARS-CoV-2-related arthritis required immunosuppressive drugs (methotrexate and sulphasalazine) [35,38].

Some patients with articular manifestations and severe lung involvement, in the context of hyperinflammatory syndrome, have been treated with IL-6 inhibitors or with Jak inhibitors, with significant improvement in both pulmonary and articular manifestations [30].

While peripheral joint symptoms have been much investigated, axial involvement related to COVID-19 is still unclear.

Novelli et al. reported a case of psoriatic spondyloarthritis triggered by SARS-CoV-2 infection and Coath and El Hasbani reported two cases of HLA-B27-positive axial spondyloarthritis that emerged closely after COVID-19 [34,38,39].

To our knowledge, our study is the first longitudinal study with MRI in post-COVID-19 sacroiliitis. Other previous case reports of sacroiliitis documented by MRI after COVID-19 included cases of seronegative spondyloarthritis, all fulfilling the classification criteria for psoriatic arthritis or axial spondyloarthritis [34,38,39].

Our patients complained of typical inflammatory lower back pain after the resolution of infectious symptoms, with clinical features of acute sacroiliitis (insidious onset, night pain, morning stiffness, improvement with exercise and NSAIDs, radiation to buttocks), as described after different infections or in the context of reactive arthritis or seronegative spondyloarthritis [41]; other differential diagnoses were excluded by personal and familiar history and laboratory tests. Both the patients were negative for HLA-B27. Sacroiliitis was then confirmed by MRI, that showed bone marrow oedema in typical sites of sacroiliac joints, and the patients responded well to NSAIDs. Even if it is well known that bone oedema can be seen in a substantial fraction of healthy people and that our patients could be at risk for developing lower back pain and even MRI abnormalities due to their work, the pre-test probability made the MRI findings more likely to be true rather than false positive [42].

On the other hand, sometimes MRI remains positive after clinical resolution in seronegative spondyloarthritis [41,43].

Whether persistence of bone marrow oedema represents the persistence of inflammatory activity (or its chronicity), or is a result of previous inflammation that requires more time to disappear, is unclear. Follow-up of those patients is ongoing to monitor both clinical and imaging evolution.

Generally, imaging findings are not specific, since X-rays are negative and ultrasound can reveal synovitis, enthesitis or tenosynovitis. Data on MRI are scarce, since only three other cases have studied sacroiliitis with MRI [34,38,39].

The exact mechanism through which COVID-19 might cause arthropathies is not completely understood.

Most authors assume the existence of molecular mimicry between viral epitopes and the synovial membrane causing local inflammation; other theories speculate about the presence of circulating immune complexes or possible localisation of the virus directly on joint tissue [14,44].

Interestingly, a mechanism involving molecular mimicry of heat shock proteins/molecular chaperones, similar to that described in immune adverse events secondary to immunotherapy for cancer, could be hypothesised. In fact, tumour cells can display on their surface heat shock proteins/molecular chaperones that are mimicked by SARS-CoV-2 molecules. Molecular mimicry between SARS-CoV-2 and tumoural proteins can elicit an abnormal immune reaction by cytotoxic cells against the virus that cross-react with the tumour cells [45].

In reactive arthritis, chlamydial heat shock protein 60 mimics host self-proteins and thus can contribute to initiation and maintenance of autoimmune/autoinflammatory diseases [46].

Heat shock proteins have been repeatedly implicated in the pathogenesis of rheumatoid arthritis, and increased Hsp27 and Hsp90α mRNA levels have been found in rheumatoid arthritis synovial tissues [47,48].

At the moment, the relationship with HLA-B27 positivity is not clear for COVID-19-related arthritis, while its influence on reactive arthritis is well known [49,50,51].

Predisposing factors to develop articular involvement consequent to SARS-CoV-2 infection are still unknown, but by evaluating medical literature, it seems that longer persistence of the virus, as demonstrated by the protracted positivity of nasopharyngeal swab for SARS-CoV-2, and its spreading from the respiratory tract to other sites including the gastrointestinal tract, might locally activate immunological and inflammatory pathways that could lead to the development of arthritis in some patients, including seronegative spondyloarthritis, which has been linked to an altered intestinal microbiome [52,53].

Moreover, as described in many studies, SARS-CoV-2 is able to activate in different ways the immune system; in particular, it can induce interleukin-6 (IL-6)-dependent pathways leading to cytokine storm and macrophage activation syndromes; in addition, it might alter interferon signalling in some cases [19,54,55,56,57,58].

Those altered inflammatory systems may elicit autoimmune processes in predisposed individuals.

A mild elevation of some cytokines was identified in our patients; in particular, IL2R, TNFalpha and IL8, which have been clearly linked both to COVID-19 and inflammatory arthritis [59].

Despite negativity of specific antibodies and different clinical features, COVID-19 shares similarities with rheumatoid arthritis, in particular regarding the cytokine pattern involved in the inflammatory process, so inhibitors of these molecules could be used in selected groups of patients, as used for the treatment of rheumatoid arthritis [19,55,60,61]. Nevertheless, endemic human coronaviruses seem to be associated with an increased risk of developing rheumatoid arthritis, as described for other viral agents such as parainfluenza virus and metapneumovirus [62]; therefore, the SARS-CoV-2 pandemic might potentially lead to increasing cases of rheumatoid arthritis in the near future [63,64,65].

Hopefully, when an antiviral therapy is available, patients with persistent positivity of SARS-CoV-2 on nasopharyngeal swab, even if asymptomatic, could be treated to obtain a faster virus clearance, since the occurrence of post-infectious manifestations may be decreased by reducing the spread and the exposure of the human immune system to the virus.

## Figures and Tables

**Table 1:** Demographic and clinical characteristics of the two patients with reactive sacroiliitis post COVID-19 infection.

Feature	Patient 1	Patient 2
Age, year	58	53
Gender	Female	Female
Comorbidity	Autoimmune hypothyroidism	Autoimmune hypothyroidism
First COVID-related symptoms	End of March	End of March
First sacroiliitis symptoms	End of April	End of April
Improvement of peripheral pain and inflammatory back pain	End of May	End of May
Nasopharyngeal SARS-CoV-2 POSITIVE buffer (date, month/day)	03/27, 04/04, 04/07, 04/17	03/27, 04/10, 04/17
Nasopharyngeal SARS-CoV-2 NEGATIVE buffer (date)	04/24, 04/28, 05/12, 05/19	04/24, 04/28, 05/12, 05/19
Drugs for the infection	HCQ and azithromycin	HCQ and azithromycin
Treatment of the sacroiliitis	NSAIDs	NSAIDs
Clinical outcome	Recovery	Recovery
First MRI	June 2020, bilateral sacroiliitis	June 2020 unilateral sacroiliitis
Second MRI	July 2020, bilateral sacroiliitis	July 2020 unilateral sacroiliitis
Third MRI	January 2021, bilateral sacroiliitis	February 2021, unilateral sacroiliitis

Legend: NSAID, non-steroidal anti-inflammatory drug; HCQ, hydroxychloroquine; MRI, magnetic resonance imaging.

**Table 2:** Laboratory features at sacroiliitis diagnosis and during follow-up at our centre.

LABORATORY FEATURES	Patient 1			Patient 2		
	First Visit (June 20)	Second Visit (July 20)	Last Visit (March 21)	First Visit (June 20)	Second Visit (July 20)	Last Visit (March 21)
Serology (UA/mL) (Negative < 8; Borderline 8–12; Positive > 12)	IgG 65.4 UA/mL IgM 2.76 UA/mL	IgG 75 UA/mL, IgM 12.4 UA/mL	ND	IgG 32.2 UA/mL, IgM 1.9 UA/mL	IgG 17.3 UA/mL, IgM 0.9 UA/mL	ND
Fibrinogen, mg/dL (normal range 180–380)	248	295	250	272	406	247
CRP, mg/L (normal range 0–5.00)	1.20	2.24	1.41	1.49	4.56	2.61
IL1 beta, pg/mL (normal range < 0.001)	0.	0.	ND	0.	0	ND
IL6, pg/mL (normal range < 7)	0.9	2	2	1.3	2	2
IL2R, pg/mL (normal range 440–1435)	1346	1446	ND	904	835	ND
IL8, pg/mL (normal range 6.7–16.2)	12.6	13.9	ND	19.2	53	ND
IL10, pg/mL (normal range 1.8–3.8)	1.8	2	ND	2.1	2	ND
TNF alpha, pg/mL (normal range 7.8–12.2)	8.9	13.2	ND	11	66	ND
IFN gamma, pg/mL (normal range < 0.99)	0	1.3	ND	0	0	ND
IP10, pg/mL (normal range 37.2–222)	112	127	ND	90.6	96	ND
Haemoglobin, g/dL	13.7	13.4	13.9	13.8	13.1	13.3
Leucocyte count, cell/mm^3^	3970	4050	4400	3960	4970	5070
Platelet count, cell/mm^3^	197,000	223,000	202,000	321,000	305,000	357,000
Antinuclear antibody (ANA)	Negative	ND	Negative	Negative	ND	Negative
Anti-SSA/SSB	Negative	ND	ND	Negative	ND	ND
Rheumatoid factor (RF)	Negative	ND	ND	Negative	ND	ND
CPK, IU/L (normal range 26–170)	187	266	171	101	147	102

Legend: CRP, C-reactive protein; IL, interleukin; IL2R, interleukin 2 receptor; IFN, interferon; TNF, tumour necrosis factor; IP10, human interferon inducible protein 10; CPK, creatine phosphokinase; ND, not done.

**Table 3:** Case reports of SARS-CoV-2-related arthritis.

	Sex and Age	Onset	Articular Involvement	SF Analysis	Presence of Crystals	SF Culture	SF RT-PCR for SARS-CoV2	RF and/or ACPA	ANA	HLA-B 27	Synovial Biopsy	Imaging	Therapy	Outcome
Talarico et al., 2020 [13]	M 45	at COVID-19 diagnosis	polyarticular (bilateral MCP and PIP, right wrist)	NA	NA	NA	NA	neg	NA	NA	NA	US: slight effusion of the involved joints	oral steroid treatment	arthralgia after steroid suspension
Gasparotto et al., 2020 [14]	M 60	32 days after COVID-19 diagnosis	right knee and ankle	yes	no	neg	neg	neg	neg	neg	NA		oral NSAIDs	recovered
Liew et al., 2020 [15]	M 47	at COVID-19 diagnosis	right knee	yes	no	(neg GRAM stain)	neg	NA	NA	NA	NA	X-rays: no erosion	oral NSAIDs and intra-articular steroid	long-term data not available
Yokogawa et al., 2020 [16]	M 57	15 days after diagnosis	right knee	yes	no	NA	neg	neg	neg	neg	NA	NA	no therapy	recovered
Di Carlo et al., 2020 [17]	M 55	4 weeks after COVID-19 infection	right ankle, subtalar joint synovitis	NA	NA	NA	NA	NA	NA	neg	NA	US: subtalar joint synovitis	oral steroid treatment	recovered
Ono et al., 2020 [18]	M 50	21 days after COVID-19 diagnosis	left and right ankle	yes	no	neg	NA	neg	neg	neg	NA	X-rays: no erosion	oral NSAIDs and intra-articular steroid	moderate improvement, long-term data not available
Jali et al., 2020 [20]	F 39	3 weeks after infection	DIP and PIP bilateral hands	NA	NA	NA	NA	neg	neg	NA	NA	X-rays: normal	oral NSAIDs	recovered
Danssaert et al., 2020 [21]	F 37	12 days after COVID-19 diagnosis	tendonitis of the ll, lll, lV right hand extensor	NA	NA	NA	NA	neg	neg	NA	NA	MRI inflammation around the extensor involved	topical NSAIDs, oral opioid gabapentin	improvement without complete recovery, long-term data not available
Fragata et al., 2020 [22]	F 41	4 weeks after COVID-19 symptoms	polyarticular (small joints of the right hand)	NA	NA	NA	NA	neg	neg	NA	NA	NA	oral NSAIDs and oral prednisolone	recovered
Langhoff Hønge et al., 2020 [24]	M 53	4 days after hospital discharge for severe respiratory insufficiency	polyarticular (right knee bilateral ankles, lateral side of the left foot)	yes	no	neg	NA	neg	NA	neg	NA	X-rays: fluid accumulation on the right knee	oral NSAIDs and oral prednisolone	recovered
Saricaoglu et al., 2020 [27]	M 73	subacute	left l MTP, PIP, right ll PIP, DIP	NA	NA	NA	NA	neg	neg	NA	NA	X-rays: no erosion	oral NSAIDS	recovered
Parisi et al., 2020 [29]	F 58	25 days after COVID-19 symptoms	ankle arthritis	NA	NA	NA	NA	neg	neg	neg	NA	NA	oral NSAIDs	recovery from symptoms, US still positive after one month
Alivernini et al., 2020 [30]	M 61	at COVID-19 diagnosis	polyarticular	yes	no	NA	NA	neg	neg	NA	Inflammatory infiltrates in the synovia	US: synovitis	baricitinib and oral prednisone	recovered
De Stefano et al., 2020 [31]	M 30	10 days post COVID-19 symptoms resolution	right elbow	yes	no	NA	NA	neg	neg	neg	NA	US: synovitis PD+	oral NSAIDs	recovered
Schenker et al., 2020 [32]	F 65	10 days after COVID-19 symptoms cessation	polyarticular (bilateral ankles knee and wrists joints)	NA	NA	NA	NA	neg	neg	pos	NA	NA	prednisolone	immediate steroid response, follow-up not available
Salvatierra et al., 2020 [33]	F 16	3 weeks after COVID-19 symptoms	II, IV and V left toes dactylitis	NA	NA	NA	NA	neg	neg	neg	NA	NA	oral NSAIDs	recovered
Shokraee et al., 2021 [36]	F 58	2 weeks after COVID-19 symptoms initiation	right hip	NA	NA	NA	NA	NA	NA	NA	NA	US: articular effusion and synovium thickness MRI: fluid rim	oral NSAIDs and prednisolone	recovered
Cincinelli et al., 2021 [37]	M 27	2 weeks after COVID-19 diagnosis	I MCF	NA	NA	NA	NA	NA	NA	NA	NA	NA	oral NSAIDs and oral prednisolone	recovered
El Hasbani et al. (case 2) [38]	M 57	One month after COVID-19 infection	left wrist	NA	NA	NA	NA	neg	neg	pos	NA	MRI: wrist synovitis	oral prednisolone and oral NSAIDs	recovered

Legend: M, male; F, female; SF, synovial fluid; RF, rheumatoid factor; ACPA, anti-citrullinated protein/peptide antibody; ANA, antinuclear antibody; Neg, negative; DIP, distal interphalangeal joint; MTP, metatarsophalangeal; NSAIDs, non-steroidal anti-inflammatory drugs; PIP, proximal interphalangeal joint; PD, power doppler; US, ultrasound; NA, not available.

## Data Availability

All data available are reported in the text, tables and figures.
